# Rhamnose-Inducible Gene Expression in *Listeria monocytogenes*

**DOI:** 10.1371/journal.pone.0043444

**Published:** 2012-08-22

**Authors:** Lars Fieseler, Sibylle Schmitter, Justinas Teiserskas, Martin J. Loessner

**Affiliations:** Institute of Food, Nutrition, and Health, ETH Zurich, Zurich, Switzerland; University of Illinois at Chicago College of Medicine, United States of America

## Abstract

Acid production from rhamnose is a characteristic phenotype of *Listeria monocytogenes*. We report the identification of the rhamnose transport and utilization operon located at *lmo2846* to *lmo2851*, including the rhamnose-dependent promoter P*_rha_*. Expression of reporter genes under control of P*_rha_* on a single copy integration vector demonstrated its suitability for inducible gene expression in *L. monocytogenes*. Transcription initiation from P*_rha_* is dose dependent, and a concentration as low as 100 µM rhamnose was found sufficient for induction. Moreover, P*_rha_* is subject to glucose catabolite repression, which provides additional options for strict control of expression. Infection of human THP1 macrophages revealed that P*_rha_* is repressed in intracellular *L. monocytogenes,* which is explained by the absence of rhamnose in the cytosol and possible interference by catabolite repression. The P*_rha_* promoter provides a novel and useful tool for triggering gene expression in extracellular *L. monocytogenes*, whereas intracellular conditions prevent transcription from this promoter.

## Introduction

*Listeria monocytogenes* is a non-sporulating Gram-positive rod, and the causative agent of human Listeriosis, a severe infection transmitted via contaminated food. The bacterium serves as a model organism for studies in cellular microbiology, bacterial pathogenicity and virulence, bacteriophage biology, and food safety [1], [2], [3], [4].

*L. monocytogenes* is able to produce acid from rhamnose, whereas *L. innocua*, *L. welshimeri*, and *L. grayi* show variable rhamnose utilization, and strains of *L. ivanovii*, *L. seeligeri*, and *L. marthii* are negative [5], [6]. Rhamnose is a naturally occurring L-6-deoxy hexose. It is present as a substituent of pectin in plant cell walls where it is periodically attached via α-1, 2-glycosylic linkages to galacturonic acid. In *L. monocytogenes* serovar 1/2 strains, rhamnose is not only used as a carbon source, it is also found as a decoration of the cell wall teichoic acids, and required for adsorption of A118 like bacteriophages [7].

**Figure 1:**
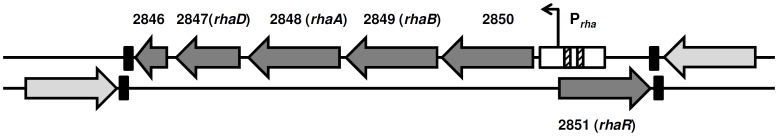
The *L. monocytogenes* rhamnose utilization operon. Dark grey: open reading frames *lmo2846-lmo2851* located on both DNA strands [9] and their functional assignments; white: P*_rha_* promoter, putative -10 and -35 regions are shaded; black: transcription terminators.

In *Listeria,* metabolism of rhamnose results in production of 1,2-propanediol, which is further oxidized to propionate [8]. *In silico* analysis indicated that rhamnose catabolism in both *L. monocytogenes* and *L. innocua* correlates with the presence of a set of genes clustered in a rhamnose operon (*lmo2846-lmo2850* and *lin2978-lin2982,* respectively) [9], which encodes an ABC-transporter, a rhamnulokinase, rhamnose isomerase, rhamnulose-1-phosphate aldolase, and a rhamnose epimerase. An AraC-type DNA binding transcriptional regulator (*lmo2851* and *lin2983,* respectively) is located immediately adjacent (Fig. 1). While the function of rhamnose operon genes and products has not been experimentally confirmed in *Listeria*, the specific uptake of rhamnose and the molecular biology and transcriptional regulation of these genes and their products has been thoroughly studied in *E. coli*. Remarkably, the structure and composition of the *E. coli rha* operon differs considerably from *L. monocytogenes*. While a rhamnose epimerase is not present, *E. coli* encodes two AraC-type regulatory proteins, RhaS and RhaR, to control *rhaBAD* expression [10], [11], [12], [13], [14], [15].

Gene products involved in carbohydrate transport and metabolism are often highly conserved yet specific for different prokaryotes. While *E. coli* encodes several carbohydrate transporters, e.g., the *rha*-, *ara*- and *lac* operons, facilitating rhamnose, arabinose and lactose uptake, respectively, *B. subtilis* features the *xyl* operon for xylose utilization. Gene expression and synthesis of carbohydrate transporters and accessory proteins is strictly regulated, and often depends on glucose-mediated catabolite repression [16], [17]. In this case, gene expression (i.e., repressor inactivation) only occurs when glucose is either not present or removed from the growth medium and replaced by the respective carbohydrate. Such tight control of promoter activity by repressor proteins can be elegantly harnessed for the design of inducible gene expression systems and vectors [18], [19].

With respect to *L. monocytogenes,* inducible gene expression is a highly desirable tool to study not only its pathogenicity and virulence, but also to better understand the environmental growth properties and responses of this opportunistic pathogen. Until now, inducible gene expression in this organisms has been based upon use of the *lacI* repressor and isopropyl-β-D-1-thiogalactopyranoside (IPTG) as an inducer [20], [21]. A disadvantage of this system is its poor stringency and often high background expression when a strong promoter is used. In fact, poor tune-ability and high level of read-through transcription can make it difficult or impossible to obtain quantitative data, or to clone or express toxic genes (L. Fieseler and M. J. Loessner, unpublished data). In contrast, positively-regulated gene expression (e.g., by an arabinose or rhamnose-dependent DNA binding AraC-type repressor) responds much slower following induction, while transcription repression is much tighter compared to negatively-regulated systems such as the *lac* operon [22].

Therefore, the aim of this study was to develop a tightly regulated, rhamnose-inducible gene expression system for *Listeria,* based on the P*_rha_* promoter. To monitor transcription activity, reporter genes encoding green fluorescent protein and drug resistance were placed under control of P*_rha_* on a single-copy integration vector. Employing both *in vitro* growth and intracellular infection models, we demonstrate that P*_rha_* enables quantitative expression of target genes, which can be modulated by the presence and concentration of rhamnose.

## Results

### Identification of the *L. monocytogenes* P*_rha_* Promoter

**Figure 2:**
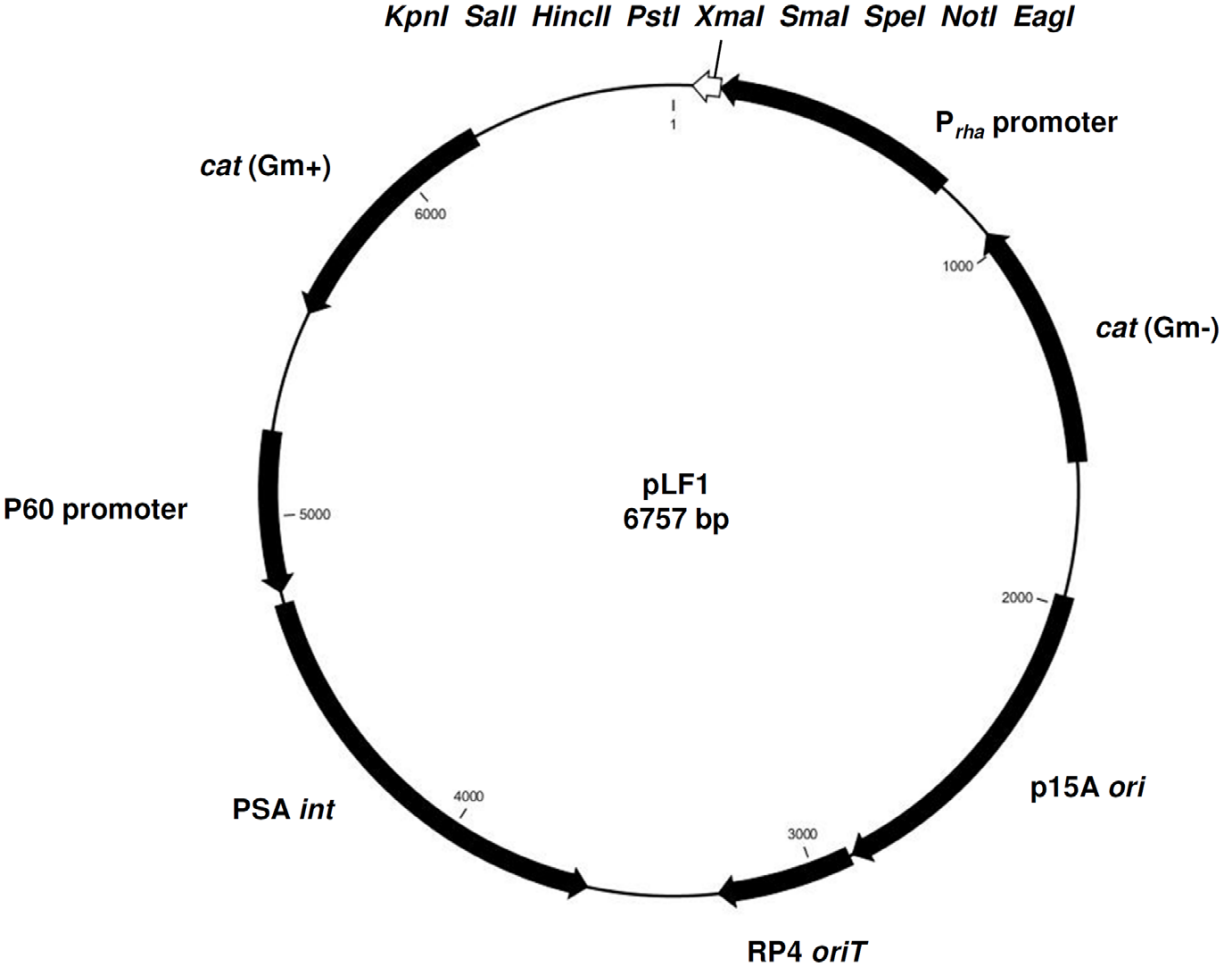
Plasmid pLF1 for rhamnose-dependent gene expression in *Listeria*. The P*_rha_* promoter sequence was inserted into the multiple cloning site of the single copy integration shuttle vector pPL2 [35]. The indicated restriction sites *Eag*I, *Not*I, *Spe*I, *Sma*I, *Xma*I, *Pst*I, *Hinc*II, *Sal*I, and *Kpn*I remain available for downstream insertions.

Putative -10 and -35 regions of a promoter designated as P*_rha_* were identified *in silico* immediately upstream of *lmo2850*, the first gene of a putative rhamnose utilization operon in *L. monocytogenes* (Fig. 1) [9]. In order to be able to test P*_rha_* for inducible gene expression, a 671 bp DNA fragment was amplified from the region upstream of the Shine-Dalgarno sequence of *lmo2850* (nucleotide positions 2939946–2940597), and inserted into plasmid pPL2 to yield pLF1 (Fig. 2). Downstream of P*_rha_*, pLF1 still features the very useful multiple cloning site from pPL2, i.e., unique *Eag*I, *Not*I, *Spe*I, *Sma*I, *Xma*I, *Pst*I, *Hinc*II, *Sal*I, and *Kpn*I sites available for insertions and cloning.

### Transcription from P*_rha_* in *Listeria* is Rhamnose-dependent

**Figure 3:**
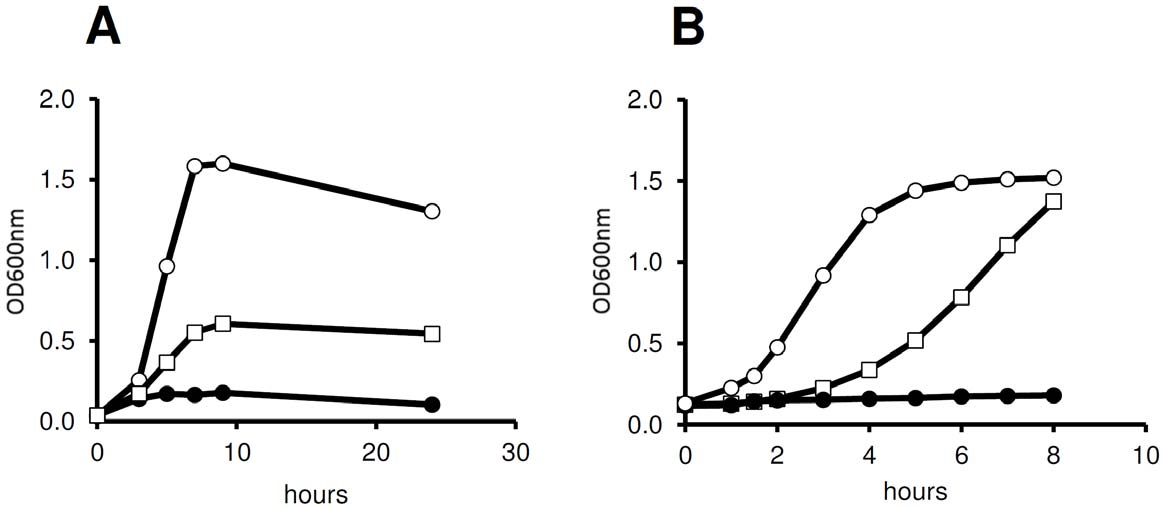
Rhamnose dependent growth of *L. monocytogenes* in batch culture. Panel A: Growth of *L. monocytogenes* 10403S in LB broth supplemented with glucose (50 mM, open circle) and rhamnose (50 mM, open squares), respectively. When no carbon source was added (closed circles), *L. monocytogenes* showed only poor growth. Panel B: Growth of *L. monocytogenes* LF002 (*ermC* under control of P*_rha_*) in medium supplemented with 7.5 µg/ml erythromycin. LF002 cells pre-induced with rhamnose (open squares) showed a slight delay in growth response, whereas non-induced cells (closed circles) did not multiply. *L. monocytogenes* strain LF003 (constitutive *ermC* expression) was used as positive control (open circles).

*L. monocytogenes* is able to utilize rhamnose (50 mM) as a carbon source during growth (Fig. 3A). Cells entered log phase after approximately 2 h, entered stationary phase after approximately 7 h, featuring an optical density (OD_600 nm_) of approximately 0.6. In the positive control (50 mM glucose), the culture reached a higher maximum OD_600 nm_ of 1.6 after 7 h incubation, while only poor growth was observed in LB broth without additional carbon source (negative control).

To determine whether expression from P*_rha_* is actually rhamnose-dependent, *ermC* was used as a reporter. *L. monocytogenes* LF002 was incubated in the presence of 10 mM rhamnose, before addition of 7.5 µg/ml erythromycin to the culture. While non-induced bacteria did not multiply further, rhamnose-induced cells continued growth and reached an OD_600 nm_ of 1.4 after 8 h of incubation (Fig. 3B). The positive control (*L. monocytogenes* LF003 with constitutive expression of *ermC*) featured a shorter lag phase and faster onset of growth, and reached the stationary phase after 6 h of incubation (OD_600 nm_ of 1.5).

**Figure 4:**
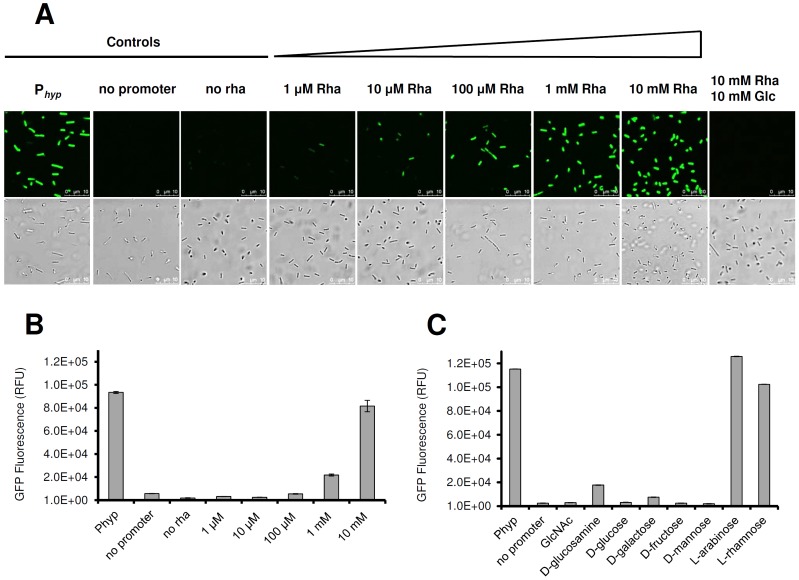
Rhamnose-inducible expression of GFP. Panels A and B: Dose-dependent response of P*_rha_*-controlled *gfp* expression in *L. monocytogenes* LF001 after 16 h of induction using rhamnose concentrations as indicated. Panel A, top row: fluorescence microscopy; bottom row, phase contrast microscopy. Positive and negative controls, and the effect of catabolite repression by addition of equimolar amounts of glucose and rhamnose are indicated. rha: rhamnose, glc: glucose. Panel B: quantitation of relative fluorescence (RFU) of GFP in rhamnose-induced bacteria. Positive (P_hyp_) and negative (no promoter) controls are indicated on the left. Addition of 100 µM rhamnose increased fluorescence significantly (p<0.001). Panel C: quantitative catabolite repression of P*_rha_*-dependent *gfp* expression in the presence of rhamnose together with a second carbohydrate (indicated on the x-axis), at equimolar concentration (10 mM). Positive (P_hyp_) and negative (no promoter) controls are indicated on the right.

As an alternative to drug selection, the *gfp* gene was employed to measure expression from P*_rha_* in strain LF001, by monitoring green fluorescence following dose-dependent rhamnose induction. As negative control, LF004 (no promoter*)* was used, and LF005 (constitutive *gfp* expression from P*_hyp_*) served as positive control. We show that a concentration of 100 µM rhamnose was sufficient to quantitatively induce P*_rha_*-dependent transcription in a majority of cells (Fig. 4, panels A and B). Increasing rhamnose concentration of up to 10 mM resulted in stronger fluorescence, indicating that induction is dose-dependent. Even higher rhamnose concentrations did not result in a further increase of fluorescence, indicating that a plateau was reached. Interestingly, the presence of 10 mM glucose, N-acetylglucosamine, glucosamine, galactose, mannose, or fructose in addition to 10 mM rhamnose completely repressed P*_rha_* activity, demonstrating that transcription of the *lmo2846-lmo2850*rhamnose operon is subject to a strict catabolite repression. Interestingly, arabinose was the only sugar tested that did not affect P*_rha_* activity (Fig. 4C).

### P*_rha_* is Repressed in Intracellular *L. monocytogenes*

It was interesting to determine if rhamnose would also be useful for regulating gene expression in intracellular *L. monocytogenes* during infection and the intracellular state. In the human intestine, rhamnose is not digested and regarded as a soluble dietary fiber material. While rhamnose as an osmolyte may affect cultured mammalian cells at higher concentrations, the amounts required for activation of P*_rha_* are quite low. We did not observe any negative effect on viability when THP1 macrophages [23] were exposed to 100 mM rhamnose over a period of 40 h. Cells maintained their shape and remained attached to the bottom of the cell culture flask, indistinguishable from controls without rhamnose (data not shown).

**Figure 5:**
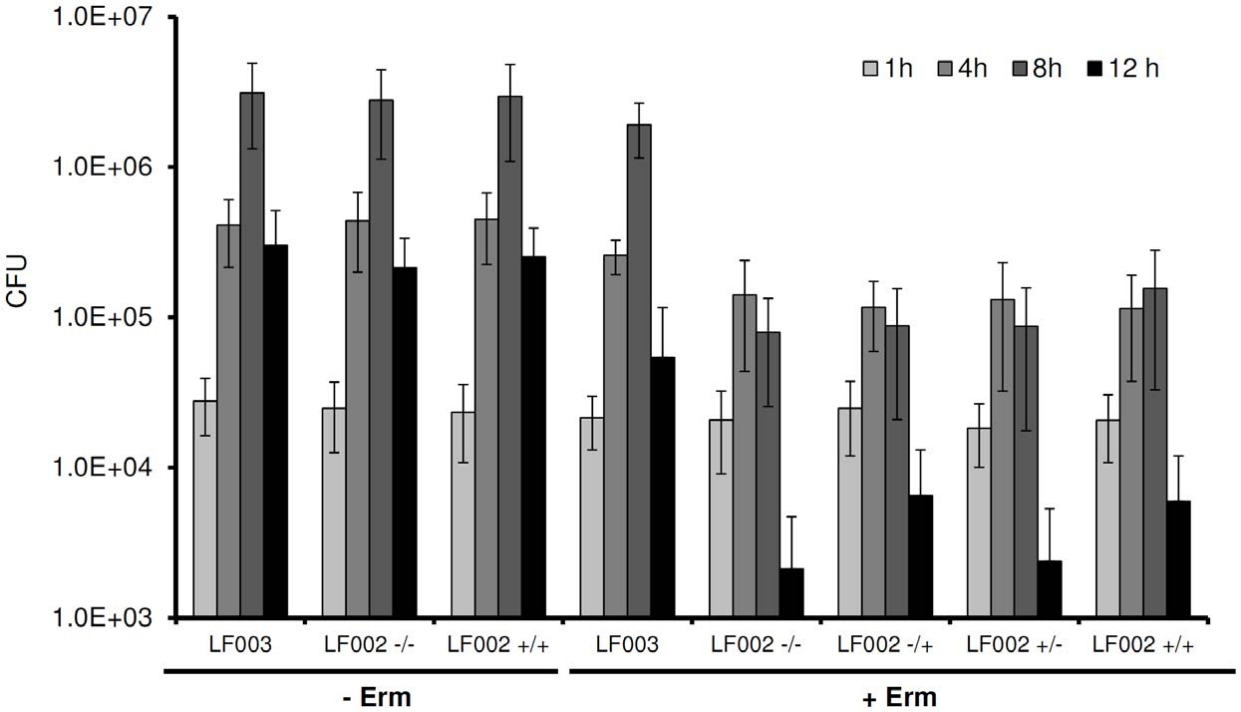
Intracellular multiplication of *L. monocytogenes* LF002 (*ermC* under control of P*_rha_*) in human THP1 macrophages, in the presence and absence of rhamnose and erythromycin (see Material and Methods for experimental details). Positive controls without drug addition (− Erm) are in the left columns. Right columns: erythromycin added to infected cells (+ Erm). –/–: non-induced bacteria, no addition of rhamnose after macrophage infection; –/+: non-induced bacteria, 10 mM rhamnose added after infection; +/–: pre-induced bacteria, no addition of rhamnose after macrophage infection; +/+: pre-induced bacteria, 10 mM rhamnose added after infection; LF003: constitutive expression of *ermC*.

To determine transcriptional activity from P*_rha_* in *L. monocytogenes* during infection, strain LF002 that features *ermC* under control of P*_rha_* was used. We found that neither induced nor non-induced bacteria were able to multiply intracellularly in infected THP1 macrophages in presence of erythromycin (Fig. 5). Moreover, addition of rhamnose to infected macrophages did not result in *ermC* expression, i.e., the intracellular bacteria were unable to grow and multiply in the presence of the antibiotic. ANOVA analyses showed that the corresponding intracellular cell counts did not differ significantly from each other. In contrast, strain LF003 with constitutive expression of *ermC* was not affected, and significantly increased by 1.8 logs after an 8 h infection period (p<0.0001). In the absence of the drug, all strains showed identical growth responses regardless if exposed to rhamnose or not (Fig. 5).

**Figure 6:**
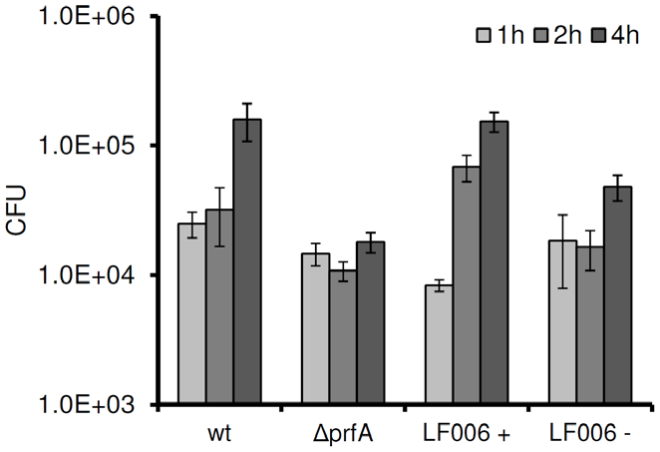
Intracellular multiplication of *prfA*-negative and P*_rha_*-dependent trans-complemented *L. monocytogenes* after infection of human THP1 macrophages. wt: wild type; Δ*prfA*: *prfA* knock out; LF006 +: single copy *prfA* under control of P*_rha_* (pre-induced overnight with 10 mM rhamnose); LF006 −: non-induced.

*L. monocytogenes* strain LF006 features a chromosomal Δ*prfA* deletion [24] of the gene encoding the regulator PrfA, which is complemented by *prfA* under P*_rha_* control on a single copy pLF6 insertion. When LF006 was pre-induced with 10 mM rhamnose prior to infection of THP1 macrophages, virulence could be fully restored (Fig. 6). Non-induced LF006 did not significantly differ in intracellular multiplication from the negative control (Δ*prfA*) one and two hours post infection. However, four hours post infection a slight increase in intracellular cell counts was observed (p<0.0001).

## Discussion

Inducible gene expression systems for bacteria are important tools for biotechnology applications and basic research. Several well defined vectors are available for model organisms such as *E. coli* or *B. subtilis* In contrast, very few systems for inducible gene expression in the opportunistic pathogen *L. monocytogenes* are available, which can be useful for tuned expression of virulence genes or genes encoding potentially toxic products. Dancz and co-workers reported an IPTG-inducible pLIV vector, where a synthetic promoter P_SPAC_ was used in combination with the *lacO*_id_ operator [20] and *lacI,* placed under control of the constitutive P_iap_ (P_60_) promoter. Similar vectors have been developed by others [25]. However, negatively regulated promoters are often leaky, due to less stringent repression and feature poor repression [22]. This renders cloning of potentially lethal or toxic genes difficult or even impossible. Because transcriptional control (i.e., repression) in positively regulated promoters such as P*_ara_*, P*_xyl_*, or P*_rha_* generally is much tighter, our goal was to design such a system for *Listeria*.

The P*_rha_* promoter identified and used in this work is silent in the absence of the sugar, and can be specifically induced in a dose dependent manner by addition of 10–100 µM rhamnose to growing cells. All strains of *L. monocytogenes* can utilize rhamnose as a single carbon source; additional P*_rha_*-binding regulatory proteins are not required. This renders application of P*_rha_* simple and straightforward, the promoter may be fused to any gene of interest. In addition, transcription is subject to catabolite repression and the promoter is not active when glucose is present, which provides additional options for P*_rha_*-controlled gene expression. However, the exact mechanism of catabolite repression is not clear. It is possible that it is similar to the situation in the maltose and maltodextrin utilization gene clusters, where catabolite expression is independent of the catabolite control protein CcpA, and likely regulated by inducer exclusion [26].

During infection of macrophages, intracellular bacteria featuring P*_rha_*-controlled *ermC* expression were unable to multiply. This can be explained by the fact that rhamnose is not actively transported over the mammalian (macrophage) cytoplasmic membrane [27], which should effectively prevent induction of P*_rha_* in intracellular bacteria. Because rhamnose is generally not available in the cytosol of human or animal cells, P*_rha_* may be employed to shut down gene expression upon entry of *L. monocytogenes* into a host cell. Moreover, catabolite repression by intracellular glucose deposits may also contribute to prevent expression from the P*_rha_* promoter. This feature can be useful for development of live attenuated vaccine vectors based on the intracellular lifestyle of this pathogen. The opposite approach, e.g., onset of gene expression after entry into the host cytosol has been realized using P*_actA_* [28]. This promoter is PrfA-dependent and specifically induced in intracellular *L. monocytogenes*. The aims were to kill and lyse intracellular *L. monocytogenes* for use as DNA delivery vehicles into eukaryotic cells, by expression of bacteriophage cell wall hydrolase *ply*_118_ under control of P*_actA_* [29].

The challenge in designing *L. monocytogenes* as a delivery vehicle for the allocation of proteins to the immune system is to attenuate the bacterium in such a way that it retains the ability to invade potential host cells, however, without causing extensive collateral damage. So far, attenuated strains often featured disruption of essential virulence factors, such as *actA*, *inlB* or *prfA* [30], [31], [32], [33]. An alternative strategy is to create *L. monocytogenes* auxotroph mutants, such as *dal* and *dat* null strains defective in D-alanine synthesis [34]. All of these strains have been applied to stimulate specific T-cell responses *in vivo*. One particular *prfA* defective strain (Lm-LLO-E7) was used as a vaccine against invasive carcinoma of the cervix in a clinical phase I study.

PrfA is a soluble cytosolic protein that controls transcription of key virulence factors in *L. monocytogenes.* We tested P*_rha_* to trigger *prfA* expression in an infection model. The *prfA*-negative phenotype could be fully complemented, but only when expression of the gene in strain LF006 was pre-induced with rhamnose prior to macrophage infection (Fig. 6). In this case, induction of *prfA* resulted in an overproduction of the protein compared to the wild type, and it is reasonable to assume that the intracellular concentration of PrfA was sufficiently high to ensure its presence and function over the next few generations, i.e., cell divisions. Hence, even when rhamnose as inducer of *prfA* expression is removed upon invasion and entry into host cells, the protein likely was present at a sufficient threshold concentration to activate transcription of the PrfA regulon in the daughter cells. Further experiments would be required to demonstrate that these cells progressively lose virulence after prolonged incubation, division and intracellular life. In fact, finely tuned *prfA* induction from P*_rha_* by lower rhamnose concentrations could yield the individually desired control of a corresponding phenotype.

In these experiments, we also noted that virulence of non-induced LF006 was not as strictly attenuated as the *prfA* negative control, which may suggest a low level background expression from the P*_rha_* promoter under these specific conditions. It is not unreasonable to assume that even very small amounts of PrfA may be sufficient to slightly elevate intracellular counts of non-induced LF006. Nevertheless, the possibility of employing P*_rha_* for attenuation of virulence genes in intracellular *L. monocytogenes* for application of the organism as live vaccine remains an attractive option.

## Materials and Methods

### Bacterial Strains and Culture Conditions

*L. monocytogenes* was cultured in half-strength Brain Heart Infusion (1/2 BHI) broth at 30° or 37°C (macrophage infection model). Chloramphenicol (10 µg/ml) or erythromycin (7.5 µg/ml) was added to the media as needed. Rhamnose or other sugars were used at different concentrations, up to a maximum of 50 mM. To determine the utilization of rhamnose by *L. monocytogenes,* cells were incubated in Luria-Bertani (LB) broth substituted with 50 mM glucose and 50 mM rhamnose, respectively. *Escherichia coli* SM10 and XL1-blue MRF’ (Stratagene) were routinely cultured in LB broth at 37°C and with the addition of chloramphenicol (10 µg/ml for XL1-blue MRF’ and 20 µg/ml for SM10) when appropriate. All strains used in this study are summarized in Table 1.

**Table 1:** Bacterial strains and plasmids used in this study.

Strain or plasmid	Relevant characteristic	Reference or Source
***E. coli***		
XL1-Blue MRF’	Δ(*mcrA*)183 Δ(*mcrCB-hsdSMR-mrr*)173 *endA1 supE44 thi-1 recA1* gyrA96 *relA1lac*[F′ *proABlacI* ^q^Z ΔM15 Tn10 (Tet^r^)]	Stratagene
SM10	λpir	R. Calendar
***L. monocytogenes***		
10403S	wild type, Stm^R^	D. Portnoy
EGDe	wild type	J. Kreft
EGDe Δ*prfA*	*prfA* deleted	J. Kreft [24]
LF001	10403S derivative, tRNA^Arg^::P*_rha_ gfp*	This work
LF002	10403S derivative, tRNA^Arg^::P*_rha_ ermC*	This work
LF003	10403S derivative, tRNA^Arg^::pPL3	This work
LF004	10403S derivative, tRNA^Arg^::*gfp*	This work
LF005	10403S derivative, tRNA^Arg^::P*_hyp_ gfp*	This work
LF006	EGDe Δ*prfA*, tRNA^Arg^::P*_rha_ prfA*	This work
**Plasmids**		
pPL2	*cat cat*; *E. coli* / *L. monocytogenes* shuttle vector; thermosensitive *ori* for *Listeria*	[34]
pPL3e	pPL2 derivative, *cat ermC gfp*; *E. coli* / *L. monocytogenes* shuttle vector;thermosensitive *ori* for *Listeria*	D. Higgins [35]
pLF1	pPL2 derivative, P*_rha_*	This work
pLF2	pPL2 derivative, P*_rha_ gfp*	This work
pLF3	pPL2 derivative, P*_rha_ ermC*	This work
pLF4	pPL2 derivative, *gfp*	This work
pLF5	pPL2 derivative, P*_hyp_ gfp*	D. Higgins
pLF6	pPL2 derivative, P*_rha_ prfA*	This work
pLEB599	Source of *ermC*	T. Takala

### Bioinformatics

The *lmo2846-lmo2850* operon in the *L. monocytogenes* genome [9] was identified and analyzed *in silico* (CLC Main Workbench software; CLC Bio, Aarhus, Denmark). Putative -10 and -35 regions were identified by a promoter-finding algorithm (BPROM, http://linux1.softberry.com/berry.phtml).

### Cloning Procedures

Plasmid pPL2 was used for cloning and single copy insertion into a tRNA_Arg_ gene via bacteriophage PSA site-specific integrase. The plasmid remains stable in the absence of drug selection, and does not cause polar effects [35].

The P*_rha_* promoter region was PCR amplified using primers P*_rha_*-f (5′- ATTGCGAGCTCTATTCCGTGATAATTTGG-3′) and P*_rha_*-r (5′- AAACGGCCGACTCATTTT AGTTAAGCGC-3′), yielding a 671 bp product. Following digestion with *SacI* and *EagI (*sites are underlined), the fragment was inserted into pPL2 to yield pLF1. The *gfp* gene was excised from pPL3 [36] (kindly provided by D. Higgins, Harvard Medical School, USA), using enzymes *EagI* and *KpnI*, purified by gel extraction, and inserted into the corresponding sites in pLF1 downstream of P*_rha_*. The *ermC* gene was amplified from pLEB579 (kindly provided by T. Takala, University of Helsinki, Finland), using primers *ermC*-f (5′-AAACGGCCGACCAAA TTAAAGAGGGTTATAATG-3′) and *ermC*-r (5′-AAAGGTACCGAAAA ACAAGTTAAGGGATGC-3′), digested with *Eag*I and *Kpn*I (sites are underlined), and cloned into pPL2. Ligation reactions were transformed into *E. coli* SM10 by electroporation, clones containing the desired inserts were identified by colony PCR, and plasmids recovered by a standard alkaline lysis method. The promoter region and inserts of interest were sequenced. Details of the plasmids and strains are listed in Table 1.

*E. coli* SM10 carrying pLF-derived plasmids were then used for vector transformation by conjugation into *L. monocytogenes* 10403S, applying a mating procedure as described previously [35]. *L. monocytogenes* EGDe was transformed using electroporation [37]. The desired clones were selected on BHI agar containing 200 µg/ml streptomycin and/or 10 µg/ml chloramphenicol, respectively, after incubation for 2–3 days at 30°C.

### Fluorescence Reporter Assays

The synthesis of GFP as a reporter for gene expression was monitored using confocal laser scanning microscopy (TCS SPE, Leica, Germany), or a fluorescence plate reader (VICTOR^3^Multilabel Counter, PerkinElmer, USA). To ensure complete and correct folding of mature GFP proteins, bacteria were harvested from overnight cultures, adjusted to an OD_600 nm_ of 1.0 per ml, and washed 3 times with phosphate buffered saline prior to analysis. For spectrophotometry, 200 µl of the suspension was transferred into a well of a black 96 well plate. Each sample was analyzed in triplicate, and the experiment was independently repeated three times. Arithmetic means and standard deviations are indicated.

### *Listeria* Infection of Macrophages

Human THP1 macrophages [23] were cultured in RPMI 1640 medium supplemented with 20% FBS (Sigma), at 37°C in an atmosphere containing 5% CO_2_. Approximately 5×10^5^ cells per well of a 24-well plate were seeded, and infected by addition of 1×10^7^ *L. monocytogenes* strain LF002 or LF006 (Tab. 1), for 1 h (MOI 20). For the infection, rhamnose-induced (10 mM rhamnose) or non-induced control bacteria were taken from overnight cultures incubated at 37°C, adjusted to the desired cell concentration, and washed three times in pre-warmed PBS before use. LF002 infected Macrophages were washed with prewarmed PBS and further incubated in RPMI 1640 medium containing 50 µg/ml gentamycin to inhibit extracellular bacteria. After 1 h of incubation, macrophages were lysed to determine the initial intracellular viable counts (colony forming units, cfu) of *L. monocytogenes* LF002. Cells were washed three times with prewarmed PBS, scraped off the bottom of each well, and resuspended and lysed by vigorous pipetting and vortexing using ice-cold 0.5% (v/v) Triton X-100. The lysates were diluted and plated for determination of *L. monocytogenes* cfu. Then, 10 mM rhamnose was added to the remaining infected cells, e.g., (in the remaining wells of the plate), and 7.5 µg/ml erythromycin was added1 h later. These cells were then also lysed using ice-cold 0.5% (v/v) Triton X-100 at different time points. The lysates were diluted and plated for determination of *L. monocytogenes* cfu from samples taken at 1, 4, 8, and 12 h post infection. Each experiment was performed in triplicate, and independently repeated three times. Means and standard deviations are indicated.

*L. monocytogenes* strain LF006 was used to complement the Δ*prfA* mutant from a gene under P_rha_ control (Tab 1). Macrophage infection and determination of intracellular cfu was carried out as described above, using rhamnose pre-induced and non-induced LF006 for infection. Gentamycin (50 µg/ml) was added to inhibit extracellular bacteria after an infection time of 1 h, but rhamnose or any other substance was not added to the infected cells. The cfu of intracellular *L. monocytogenes* were determined after 1, 2 and 4 h post infection as described above.

### Statistical Analyses

Statistical analyses were performed applying ANOVA and student’s t-test algorithms.
